# A new species of *Orthosiphon* (Lamiaceae) from Angola

**DOI:** 10.3897/BDJ.2.e1162

**Published:** 2014-07-30

**Authors:** Alan Paton

**Affiliations:** † Herbarium, Royal Botanic Gardens, Kew, London, United Kingdom

**Keywords:** Endemic species, southern tropical Africa

## Abstract

A new species of *Orthosiphon* (Lamiaceae), *Orthosiphon cinereus* A.J.Paton, sp. nov. from Angola is described and the eight species of *Orthosiphon* in Angola listed with reference to previous accounts. *Orthosiphon newtonii* Briq. is reduced to the synonymy of *Endostemon tubulascens* (Briq.) M.Ashby.

## Introduction

*Orthosiphon* is a genus of around 40 species found throughout the tropical old world and with one species in Colombia (Harley and Paton 2012). During the preparation of the account for the Lamiaceae for Flora Zambesiaca (Paton et al. 2013), a new species of *Orthosiphon* endemic to Angola was discovered from herbarium material. Flora Zambesiaca covers Mozambique, Malawi, Zambia, Zimbabwe, Botswana and the Caprivi Strip of Namibia, and five species of *Orthosiphon* are reported from this area of which four, *Orthosiphon schimperi* Benth., *Orthosiphon thymiflorus* (Roth) Sleesen, *Orthosiphon rufinervis* G.Taylor, *Orthosiphon nigripunctatus* G.Taylor occur in Angola. A checklist of Angolan species was provided by Figueiredo and Smith (2008) which lists two further endemic species: *Orthosiphon cuanzae* (I.M.Johnst.) A.J.Paton and *Orthosiphon violaceus* Briq. In addition *Orthosiphon pascuensis* G. Taylor should be recognized at species level, rather than as a synonym of *Orthosiphon rubicundus* (D.Don) Benth *sensu* Figueiredo and Smith (2008). With these seven species and the new species described here, there are eight species recognized in Angola. Figueiredo and Smith (2008) report three species from Angola which are not recognized here: *Orthosiphon rubicundus* (D.Don) Benth. is an Asian species similar in morphology to *Orthosiphon schimperi*; *Orthosiphon parvifolius* Vatke is only found in East Africa. *Orthosiphon newtonii* Briq is a synonym of *Endostemon tubulascens* (M.Ashby) Briq. and this new synonymy is formally recorded below.

## Taxon treatments

### Orthosiphon cinereus

A.J.Paton sp. nov.

http://specimens.kew.org/herbarium/K001057489

urn:lsid:ipni.org:names:77139693-1

#### Materials

- **Type status:** Holotype. **Occurrence:** recordedBy: G. Barbosa; F.Moreno 9730; **Location:** country: Angola; stateProvince: Huila; verbatimLocality: Tchivinguiro, picada da Banja; **Event:** year: 1961; month: 12; day: 21; **Record Level:** collectionID: urn:lsid:biocol.org:col:15867; institutionCode: K; source: http://specimens.kew.org/herbarium/K001057489
- **Type status:** Isotype. **Occurrence:** recordedBy: G. Barbosa; F.Moreno 9730; **Location:** country: Angola; stateProvince: Huila; verbatimLocality: Tchivinguiro, picada da Banja; **Event:** year: 1961; month: 12; day: 21; **Record Level:** collectionID: urn:lsid:biocol.org:col:14498; institutionCode: COI
- **Type status:** Isotype. **Occurrence:** recordedBy: G. Barbosa; F.Moreno 9730; **Location:** country: Angola; stateProvince: Huila; verbatimLocality: Tchivinguiro, picada da Banja; **Event:** year: 1961; month: 12; day: 21; **Record Level:** collectionID: urn:lsid:biocol.org:col:15078; institutionCode: LISC
- **Type status:** Other material. **Occurrence:** recordedBy: R. Deschamps; F. Murta; M. Da Silva 1183; **Location:** country: Angola; stateProvince: Huila; verbatimLocality: Serra do Bruco, pres de Sá da Bandeira; verbatimElevation: 1610 m; verbatimLatitude: 15°09'S; verbatimLongitude: 13°16'E; **Event:** year: 1972; month: 2; day: 21; **Record Level:** collectionID: urn:lsid:biocol.org:col:15078; institutionCode: LISC
- **Type status:** Other material. **Occurrence:** recordedBy: Centro de Botânica da Junta de Investigações do Ultramar, ex Herbário Missão de Huilla 313; **Location:** country: Angola; stateProvince: Huila; verbatimLocality: Without locality; **Record Level:** collectionID: urn:lsid:biocol.org:col:15078; institutionCode: LISC
- **Type status:** Other material. **Occurrence:** recordedBy: H. H. Johnston s.n.; **Location:** country: Angola; verbatimLocality: Without Locality; **Event:** year: 1883; month: 9; **Record Level:** institutionCode: K; collectionCode: urn:lsid:biocol.org:col:15867; source: http://specimens.kew.org/herbarium/K000938398

#### Description

**Figure 1.**
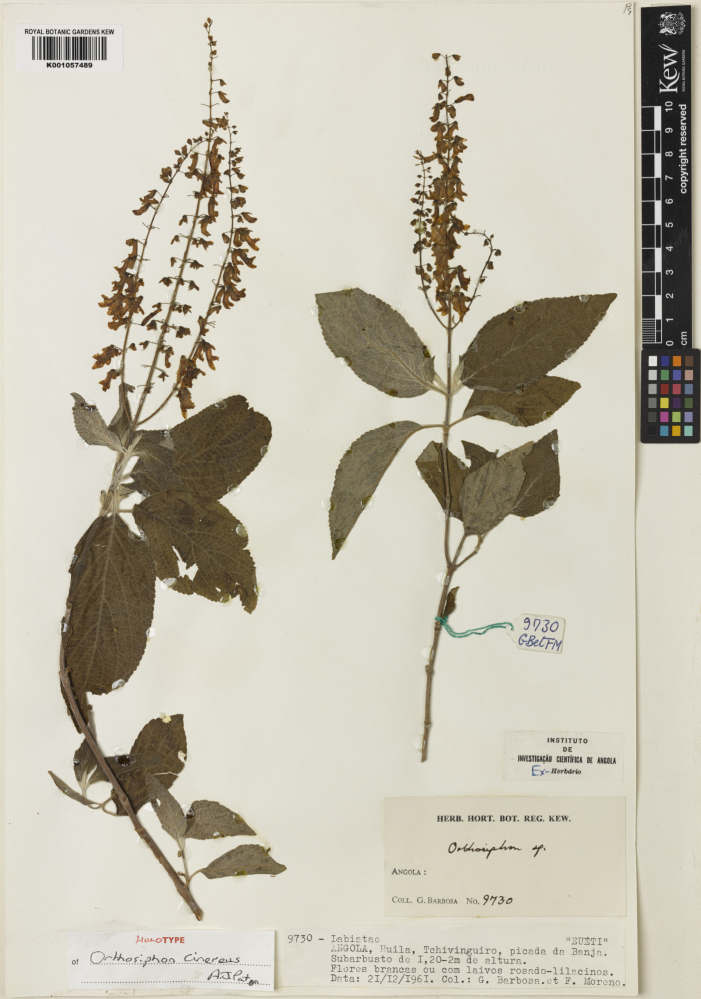
Holotype of *Orthosiphon cinereus* A.J.Paton.

Perennial subshrub 1.2–2 m tall. Stems quadrangular, pubescent, more densely so at nodes with eglandular, spreading or retrorse hairs and reddish sessile glands; inflorescence axis also with shorter glandular hairs; young shoots tomentose. Leaves petiolate; blades paler beneath, ovate, 50–120 mm long; 25–40 mm broad, shallowly serrate, base rounded to cuneate, sometimes asymmetric, pubescent with red sessile glands, grey tomentose beneath when young; petioles 8–15 mm long. Inflorescence terminal, branched at base, lax; verticils 2–6-flowered, 5–10 mm apart; bracts inconspicuous, soon deciduous; pedicels 4–5 mm long, spreading, forming a right angle with the calyx. Calyx deflexed, 4 mm long at anthesis, pubescent with reddish sessile glands; fruiting calyx 7 mm long; posterior lip erect; lateral lobes of anterior lip deltoid with posterior margin extended towards posterior lip; median lobes of anterior lip lanceolate, subulate at apex; longer than the lateral lobes. Corolla usually deflexed, pinkish, 10 mm long; tube 7 mm long, straight, dilating slightly towards throat; anterior lip cucullate enclosing stamens. Stamens 4, declinate, enclosed within the anterior lip; posterior pair inappendiculate, glabrous, adnate to the corolla above the midpoint of the tube; anterior pair glabrous, adnating nearer throat, anthers dorsifixed, synthecous. Disk 4-lobed, with anterior lobe larger. Ovary glabrous, deeply 4-lobed; style gynobasic, capitate, branches rounded, equal, adpressed. Nutlets brown, obovate with a small basal scar, minutely tuberculed, producing a small amount of mucilage when wet. Fig. 1.

#### Diagnosis

Differs from all other African species of *Orthosiphon* except *Orthosiphon thymiflorus* (Roth) Sleesen in being a shrub rather than a suffrutex sprouting annual stems, or an annual herb. Differs from *Orthosiphon thymiflorus* in being larger and more erect, 1.2–2 m tall, rather than straggling to 0.2–1.2 m tall; and with longer larger discolorous leaves, 50–120 mm long, and greyish tomentose beneath, rather than 10–40 mm long and sparsely pubescent. The flowers of *Orthosiphon cinereus* are also more strongly deflexed.

#### Etymology

Named after the grey tomentose indumentum of the stems and abaxial leaf surfaces.

#### Distribution

Endemic to Angola.

#### Ecology

Damp area in forest; 1610–1700 m, alt.

#### Conservation

No recent gatherings of this species have been collected and the specimens mostly lack precise localities. This species is best viewed as data deficient.

#### Taxon discussion

**Figure 2.**
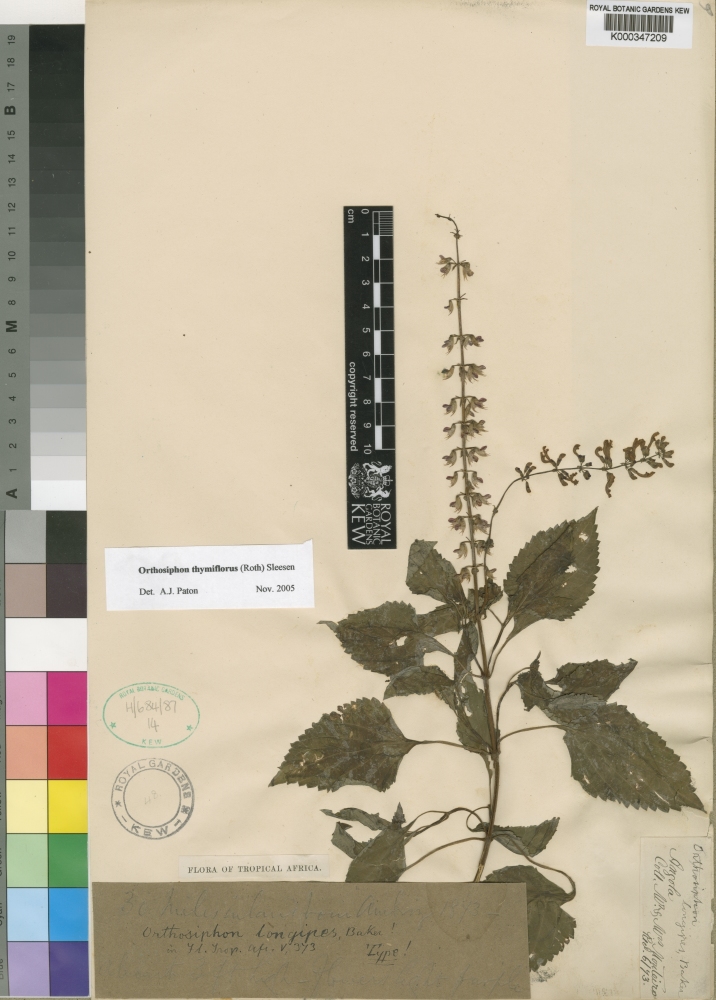
*Orthosiphon thymiflorus*. Angola, 30 miles inland from Ambriz, Monteiro, s.n., June 1843. http://specimens.kew.org/herbarium/K000347209. Holotype of *Orthosiphon longipes* Baker.

This is a distinctive, easily recognized species, differing from the most large-leaved specimens of *Orthosiphon thymiflorus* which lack the discolous, greyish leaf indumentum and have spreading rather than deflexed flowers. *Orthosiphon thymiflorus* is found throughout tropical Africa and the large leaf size seen in specimens similar to the type of *Orthosiphon longipes* Baker represent an extreme of the variation Fig. 2. *Orthosiphon longipes* was placed in synonymy of *Orthosiphon thymiflorus* in Paton et al. (2013). The shrubby habit is seen in Asia and Madagascar *Orthosiphon*, *Orthosiphon aristatus* (Blume) Miq. and *Orthosiphon adenocaulis* *A.J.Paton & Hedge* being respective examples and in *Orthosiphon americanus* Harley & A.J.Paton from Colombia, the only New World member of the genus. However, the species in Africa are mainly suffrutices with annual stems being produced from woody rootstocks. The little habitat information available from the specimens of *Orthosiphon cinereus* suggests that the species occupies a wetter forest than the seasonally dry forest habitat of *Orthosiphon thymiflorus* and other Angolan species.

### Endostemon tubulascens

(Briq.) M.Ashby

Endostemon tubulascens (Briq.) M.Ashby, J. Bot. 74: 127 (1936).
Orthosiphon tubulascens Briq., Bot. Jahrb. Syst. 19: 174 (1894).
Orthosiphon newtonii Briq., Bull. Herb. Boissier, sér. 2, 3: 990 (1903). **Synon. nov.** Holotype. Angola, Mossamedes, Humpata, Feb. 1883, F.X.O. Newton 108. http://www.herbarien.uzh.ch/static/database/details_en.php?&spTypFlg=%&spBarCod=Z-000018972&spHer=%

#### Materials

- **Type status:** Isotype. **Occurrence:** recordedBy: F. Welwitsch; individualCount: 5492; **Location:** country: Angola; stateProvince: Huilla; verbatimLocality: Morro de Lopollo; **Event:** year: 1860; verbatimEventDate: Jan to Feb; **Record Level:** collectionID: urn:lsid:biocol.org:col:15867; institutionCode: K; source: http://specimens.kew.org/herbarium/K000347216
- **Type status:** Isotype. **Occurrence:** recordedBy: F. Welwitsch; individualCount: 5492; **Location:** country: Angola; stateProvince: Huilla; verbatimLocality: Morro de Lopollo; **Event:** year: 1860; verbatimEventDate: Jan to Feb; **Record Level:** collectionID: urn:lsid:biocol.org:col:15660; institutionCode: BM

## Supplementary Material

XML Treatment for Orthosiphon cinereus

XML Treatment for Endostemon tubulascens
